# Phagotrophy in the nitrogen‐fixing haptophyte *Braarudosphaera bigelowii*

**DOI:** 10.1111/1758-2229.13312

**Published:** 2024-07-24

**Authors:** Esther Wing Kwan Mak, Kendra A. Turk‐Kubo, David A. Caron, Rachel C. Harbeitner, Jonathan D. Magasin, Tyler H. Coale, Kyoko Hagino, Yoshihito Takano, Tomohiro Nishimura, Masao Adachi, Jonathan P. Zehr

**Affiliations:** ^1^ Department of Ocean Sciences University of California Santa Cruz California USA; ^2^ Department of Biological Sciences University of Southern California Los Angeles California USA; ^3^ Marine Core Research Institute Kochi University Nankoku Japan; ^4^ Faculty of Agriculture and Marine Science Kochi University Nankoku Japan; ^5^ Fisheries Technology Institute, Japan Fisheries Research and Education Agency Hiroshima Japan

## Abstract

Biological nitrogen fixation provides fixed nitrogen for microbes living in the oligotrophic open ocean. UCYN‐A2, the previously known symbiont of *Braarudosphaera bigelowii*, now believed to be an early‐stage *B. bigelowii* organelle that exchanges fixed nitrogen for fixed carbon, is globally distributed. Indirect evidence suggested that *B. bigelowii* might be a mixotrophic (phagotrophic) phototrophic flagellate. The goal of this study was to determine if *B. bigelowii* can graze on bacteria using several independent approaches. The results showed that *B. bigelowii* grazed on co‐occurring bacteria at a rate of 5–7 cells/h/*B. bigelowii* and that the overall grazing rate was significantly higher at nighttime than at daytime. Bacterial abundance changes, assessed with 16S rRNA gene amplicon sequencing analysis, may have indicated preferential grazing by *B. bigelowii* on specific bacterial genotypes. In addition, Lysotracker™ staining of *B. bigelowii* suggested digestive activity inside *B. bigelowii*. Carbon and nitrogen fixation measurements revealed that the carbon demand of *B. bigelowii* could not be fulfilled by photosynthesis alone, implying supplementation by heterotrophy. These independent lines of evidence together revealed that *B. bigelowii* engages in phagotrophy, which, beyond serving as a supplementary source of carbon and energy, may also facilitate the indirect assimilation of inorganic nutrients.

## Graphical abstract

This research unveils that the haptophyte *Braarudosphaera bigelowii*, which contains the nitrogen‐fixing cyanobacterium UCYN‐A2, exhibits phagotrophy by grazing on co‐occurring bacteria. This discovery demonstrates a mixotrophic metabolism in this globally abundant marine nitrogen‐fixer, suggesting a combined reliance on photosynthesis and heterotrophy.

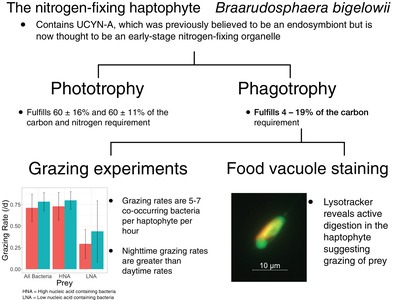

## INTRODUCTION

Phytoplankton are photosynthetic protists or cyanobacteria that are responsible for approximately half of the Earth's carbon fixation (Field et al., 1998) and form the base of the marine food web (Stoecker et al., 2017). Mixotrophy increases the efficiency of trophic transfer and thus enhances the rate of nutrient retention in the system (Fischer et al., 2017; Mitra et al., 2014; Ward & Follows, 2016). This nutritional strategy can alleviate low nutrient conditions, as phagotrophic organisms can exploit prey. Many phytoplankton are mixotrophs that combine photosynthetic (autotrophic) and heterotrophic modes of nutrition, including phagotrophy (engulfing prey or particulate organic carbon) and osmotrophy (deriving energy from dissolved organic carbon as a source of nutrients that may be unavailable to strict autotrophs) (Edwards, 2019; Rothhaupt, 1996). For example, the haptophyte *Prymnesium parvum* uses phagotrophy during phosphate‐limiting conditions (Nygaard & Tobiesen, 1993). In resource‐limited environments, having access to two types of food sources provides a competitive advantage, increasing the chances of survival.

Haptophytes are among the groups of phytoplankton that can adopt a mixotrophic mode of nutrition (Unrein et al., 2014). They are one of the most widely distributed primary producers and can account for 30%–50% of the total chlorophyll‐*a* biomass in the oceans (Liu et al., 2009). A notable example is the haptophyte *B. bigelowii* which contains UCYN‐A (*Candidatus* Atelocyanobacterium thalassa), previously believed to be an endosymbiont but now thought to be an early‐stage N_2_‐fixing organelle (Coale et al., 2024). The UCYN‐A body has now been suggested to be a nitroplast, is globally distributed (Farnelid et al., 2016; Martínez‐Pérez et al., 2016; Tang & Cassar, 2019), has a metabolically streamlined genome and lacks photosynthetic capabilities, including oxygenic photosynthesis and carbon fixation. It fixes nitrogen and provides it to *B. bigelowii* in exchange for fixed carbon (Tripp et al., 2010; Zehr et al., 2008). Both UCYN‐A and *B. bigelowii* are classified into several genotypes; UCYN‐A1 dominates the open ocean, whereas UCYN‐A2 – now understood to be the nitroplast within *B. bigelowii* (18S genotype III), is the prevalent form in coastal regions (Turk‐Kubo et al., 2017).

Previous studies suggested that the haptophyte *B. bigelowii* is also a phagotroph that grazes on bacteria. In the study by Frias‐Lopez et al. (2009), ^13^C‐labelled *Synechococcus* and *Prochlorococcus* were fed to an in situ microbial community at Station ALOHA, which is located in the North Pacific Ocean. In that study, a DNA stable isotope probing technique (DNA‐SIP) was used to identify grazers. The recovered sequences showed high similarity to two closely related sequences (97.7%–99.7% nt identity to gb|KF771254) for *B. bigelowii* and to 11 closely related sequences (97.8%–99.2% nt identity to gb|JX291836) for the haptophyte containing UCYN‐A1 based on the 18S rRNA DNA region. In another study, Krupke et al. (2015) used nanoscale secondary ion mass spectrometry (nanoSIMS) and observed an unknown structure within the haptophyte containing UCYN‐A1 which could have been an engulfed prey.

Despite the discovery of the globally important nitrogen‐fixer two decades ago, the UCYN‐A symbiosis was not isolated in culture until 2018 when *B. bigelowii* genotype III, containing UCYN‐A2, was isolated from the coastal waters of Japan (Coale et al., 2024; Suzuki et al., 2021). In the whole transcriptome analysis of the haptophyte, *B. bigelowii*, using the predictive model by Burns et al. (2018), a high likelihood of phagotrophic behaviour was indicated, with phagocyte prediction scores reaching 0.95 (Suzuki et al., 2021). The availability of the *B. bigelowii*/UCYN‐A2 culture allowed us to design experiments to directly characterize physiology, including phagotrophy. This study evaluated the mode of nutrition of *B. bigelowii* by assessing the relative contribution of phagotrophy and phototrophy during growth. Dilution experiments were also used to measure grazing rates on the bacteria in the culture and stains were used to visualize the food vacuole and assess digestive activity. Carbon and nitrogen fixation rates were also measured. Taken together, this study provides direct evidence that *B. bigelowii* is a phagotroph, which is important ecologically and offers insights into the physiological adaptations of *B. bigelowii* as a mixotrophic organism.

## EXPERIMENTAL PROCEDURES

### Culture conditions

The strain FR‐21 (NIES‐4399) established by Coale et al. (2024) was used for this study. The strain was identified through genotyping analysis. The haptophyte was identified through 18S rRNA primers as *B. bigelowii* genotype III, and the nitrogen‐fixing body was identified through nitrogenase *nifH* primers as UCYN‐A2 (Hagino et al., 2013). The cultures were maintained at a light intensity of 60 μmol photons m^−2^ s^−1^ with a light–dark cycle of 12 h:12 h at 18*°*C. For maintenance, the cultures were transferred periodically with the modified seawater‐based‐f/2 media (Guillard & Ryther, 1962) with filtered gelidium jelly extract (GJE) (Nishimura et al. 2020) as a supplement that enabled the growth of strain. The seawater medium, with a salinity of 34 ppt, used water collected from 150 and 200 m depths in Monterey Bay, California, USA (36°44′49.2″N 122°01′19.2″W). The culture was not axenic.

### Diel bacterial grazing rates determined by dilution experiments

Mortality of the co‐occurring bacterial assemblage due to the grazing by *B. bigelowii* (Strain FR‐21, NIES‐4399) was measured using the dilution method (Landry et al., 1995) in two replicate experiments. The diluent was prepared by filtering *B. bigelowii* culture through a 0.2 μm pore‐size Supor® Membrane (Pall Corp, Port Washington, NY, USA). To establish a gradient in grazing pressure, the filtered culture was then mixed with the unfiltered culture in proportions that resulted in final mixtures with 20%, 60% and 100% unfiltered culture, corresponding to 80%, 40% and 0% filtered culture, respectively. Each dilution level consisted of three replicate bottles, each of which contained a volume of 25 mL. The experiments were initiated at the beginning of the dark cycle and incubated for 24 h, following the same conditions as those used for culturing, including media, light and temperature.

Samples (2 mL) for cell counts were collected at T_0_ (start of dark cycle), T_12_ (start of light cycle) and T_24_ (end of light cycle), fixed immediately with glutaraldehyde (Electron Microscopy Sciences, Hatfield, PA, USA) for a final concentration of 0.25% v/v and incubated in the dark at room temperature for 15 min and stored at −80°C until analysis. Cell counts were obtained using a BD Influx cell sorter with a 488 nm Sapphire laser (Coherent, Santa Clara, CA, USA). All samples were diluted 10‐fold with sterile culture media to achieve the linear range of the cytometer, then filtered through a CellTrics® filter with 30 μm pore‐size mesh (Partec, Swedesboro, NJ, USA). *B. bigelowii* (grazer) was identified and gated based on the *B. bigelowii* chlorophyll‐*a* fluorescence (red fluorescence) and forward scatter (FSC; a proxy for cell size). Data collection was triggered on the FSC channel and events were counted for 5 min.

A subset of flow cytometry samples was also stained with SYBR® Green I Nucleic Acid Stain (Lonza Group, Basel, Switzerland). After incubation in the dark for 15 min, the stained bacteria within the samples were subsequently identified and counted based on the 531 nm wavelength fluorescence (green fluorescence) and FSC. Data collection was triggered on the 531 channel and events were counted for 1 min (Marie et al., 1999). The bacteria population was gated into high nucleic acid (HNA) bacteria and low nucleic acid (LNA) bacteria based on 531 nm fluorescence and side scatter (SSC). The data were processed using Flowjo 10.8.0 (Tree Star, Inc., Ashland, OR, USA).

To assess the mortality and growth rates of bacteria, changes in the measured bacterial concentrations were calculated across three sets of time points: between *t*_**0**_ and *t*_**12**_, *t*_**12**_ and *t*_**24**_ and *t*_**0**_ and *t*_**24**_ h. The growth rate for each dilution level (20%, 60% and 100%) was calculated using the natural logarithm of the ratio of final to initial bacterial concentrations. Growth rates were then plotted against the dilution series to reflect the varying degrees of grazing pressure. The mortality rate was estimated as the negative of the slope obtained by linear regression of the bacterial growth rates. In this approach, the *y*‐intercept represents the bacterial growth rate when grazers are absent (Landry et al., 1998). In the linear regression, grazing pressure was considered significant if its coefficient (the slope) had a *p*‐value <0.05.

### Measuring differential grazing of different co‐occurring bacteria

Samples were subsampled from the dilution experiment to characterize the bacterial community composition by amplification and sequencing of the 16S rRNA gene. Two‐millilitre samples were filtered through 0.2 μm pore‐size 25 mm diameter Supor filters (Pall Corp, Port Washington, NY, USA) in sterile Swinnex® holders (Millipore). The filters were transferred into 2.0 mL bead‐beating tubes that contained 0.1 and 0.5 mm diameter sterile glass beads (BioSpec Products, Bartsville, OK, USA) and stored at −80°C until further analysis. DNA was extracted using a protocol of freeze–thaw, bead‐beating and proteinase K digestion (Moisander et al., 2008) and then followed by the Qiacube® (Qiagen, Germantown, MD, USA) automated on‐column DNA extraction and clean‐up protocol using the DNeasy Plant Mini Kit (Qiagen).

To identify and quantify the bacterial community composition, the V3/V4 hypervariable region of the 16S rRNA gene was amplified using the universal primers Bakt_341F and Bakt_805R (Herlemann et al., 2011) with common sequence linkers (Moonsamy et al., 2013). The PCR amplification began with an initial denaturation step at 95°C for 5 min, followed by cycling at 95°C for 40 s, 53°C for 40 s and 72°C for 60 s for 30 cycles. The process concluded with a final elongation step at 72°C for 7 min. Libraries were prepared and paired‐end reads were sequenced using an Illumina MiSeq System at the Genomics and Microbiome Core Facility at Rush University Medical Center (Green et al., 2015). All reads were processed through quality control, denoised, merged and chimera‐checked using DADA2 and formed amplicon sequence variants (ASV) (Callahan et al., 2016). Taxonomy was assigned using the SILVA reference database (Quast et al., 2012). The R package phyloseq (McMurdie & Holmes, 2013) was used to analyse community composition and DESeq2 (Love et al., 2014) was used to calculate the differential change in the community composition of each treatment between the time points.

### *Staining* B. bigelowii *with Lysotracker™ Green*

Lysotracker**™** Green DND‐26 (Thermo Fisher Scientific, Waltham, MA, USA), a cell‐permeable green dye that stains acidic intracellular compartments (Sintes & Del Giorgio, 2010), was used to stain *B. bigelowii*. A stock solution of 1 mM Lysotracker**™** Green DND‐26 was diluted 1:20000 to stain 1 mL samples from the culture. *B. bigelowii* culture was grown to a concentration of 10^6^ cells/mL and then sampled at day and night time points. The cells were analysed immediately without any fixation by epifluorescence microscopy (Axio Imager 2, Zeiss, Dublin, CA, USA) at 630× magnification and analysed using Zeiss ZEN Digital Imaging for Light Microscopy software. The chlorophyll signal was excited using red fluorescence. The stained *B. bigelowii* was also analysed using flow cytometry, based on 531 nm fluorescence and forward scatter (FSC1). As a control, *B. bigelowii* without staining were also analysed using the same cytometry method.

### *Measurements of the growth rate, C fixation and N_2_ fixation rate of* B. bigelowii

*Braarudosphaera bigelowii* was cultivated under consistent conditions, including the same media, the light intensity of 60 μmol photons m^−2^ s^−1^ and a temperature of 18°C, maintained for more than 10 generations. The culture was diluted to the same starting concentration every 3 days to maintain the exponential phase (Figure S1) while subsampled for measurements of growth rate, C and N_2_ fixation rates at each transfer. To measure the growth rate, 0.5 mL of the culture was sampled daily in three replicate culture bottles at the same time of day, fixed immediately with glutaraldehyde (Electron Microscopy Sciences, Hatfield, PA, USA) at a final concentration of 0.25% v/v, then incubated at room temperature in the dark for 15 min. Fixed samples were then flash‐frozen at −80°C until further analysis.

All samples were diluted 10‐fold with 0.2 μm filtered culture media to achieve the linear range of the cytometer and *B. bigelowii* cells were identified and counted based on the *B. bigelowii* chlorophyll‐*a* fluorescence and forward scatter (FSC), events were counted for 5 min as described in the previous section. To determine the steady‐state growth rates (*μ*_*B. bigelowii*_) of *B. bigelowii*, we plotted the natural logarithm of the cell concentration against time to obtain a growth curve. A linear regression analysis was then applied to this growth curve and the slope of the resulting line represented the steady‐state growth rate. This approach is encapsulated by the equation:

(1) $$\mu_{B.\;\text{bigelowii}}=\ln\left(\frac{\text{Final cell concentration}}{\text{Initial cell concentration}}\right)÷t$$

Bulk C and N_2_ fixation rates were measured by the ^13^C and ^15^N_2_ tracer method of Montoya et al. (1996), amended by Mohr et al. (2010) and Wilson et al. (2012). ^15^N_2_ enriched seawater was prepared following the procedure described by Klawonn et al. (2015). Eight millilitres of ^15^N_2_ gas (99 atom %, Cambridge Scientific) were injected into 250 mL of 0.2 μm pore‐size filtered culture medium, vortexed for 5 min and stabilized at room temperature for at least 24 h. ^15^N_2_ enriched seawater was then transferred to 25 mL crimp‐sealed glass serum bottles. Three bottles were then collected from the entire batch of ^15^N_2_ enriched seawater to be used for later evaluation of the initial atom % of N_2_. This analysis was performed using Membrane Inlet Mass Spectrometry (MIMS), following the method outlined by Ferrón et al. (2016).

To start the incubation, 1 mL of ^15^N_2_ enriched seawater and 300 μL of 59 mM ^13^C‐labelled bicarbonate (final concentration: 1.17 mmol/L 99 atom% NaH^13^CO_3_, Cambridge Isotope Laboratories) were added to 14 mL of *B. bigelowii* culture in 15 mL serum bottles, crimp‐sealed and incubated at 18°C for 24 h. The T_0_ δ^13^PC /δ^15^PN natural abundance samples were prepared by adding 1 mL of filtered medium to 14 mL of *B. bigelowii* culture. After 24 h, each incubation was filtered onto pre‐combusted 25 mm diameter glass fibre filters (GF/F, Whatman) and frozen and stored at −80°C.

The filters were then dried at 60°C for 24 h, followed by acid fumigation with hydrochloric acid for 48 h and then pelleted into tin capsules. Particulate C, particulate N and isotopic composition (δ^13^C, δ^15^N) were analysed using a CE Instruments NC2500 elemental analyser coupled to a Thermo Scientific DELTAplus XP isotope ratio mass spectrometer via a Thermo‐Scientific Conflo III at the University of California Santa Cruz Stable Isotope Laboratory. N_2_ fixation rates were calculated from the following equation (Montoya et al., 1996):

(2) $$N_{2}\;\text{fixation rate}\;\left(\text{fmol}\;{\text{cell}}^{-1}d^{-1}\right)=\frac{A_{\mathrm{PN}}-A_{{\mathrm{PN}}_{0}}}{A_{N_{2}}-A_{{\mathrm{PN}}_{0}}}\times \frac{\left[\mathrm{PN}\right]}{\Delta t}\times \frac{1}{\text{cell}}$$

where *A*_PN_ and *A*_PN0_ are the fractional ^15^N enrichment (in units of atom %) after and before the incubation, respectively. [PN] is the particulate nitrogen concentration at the end of the incubation period, *t* is the duration of incubation in units of hours and *A*_N2_ is the fractional ^15^N‐enrichment of the N_2_ source pool, determined by MIMS. To calculate the N_2_ fixation rate per cell, the bulk rate was then divided by cell count, determined by flow cytometry. Limits of detection (LOD) and minimum quantifiable rates (MQR) were calculated as in Gradoville et al. (2017).

Similarly, C fixation rates were determined by the following equation:

(3) $$C\;\text{fixation rate}\;\left(\text{fmol}\;{\text{cell}}^{-1}d^{-1}\right)=\frac{A_{\mathrm{PC}}-A_{{\mathrm{PC}}_{0}}}{A_{{\mathrm{CO}}_{2}}-A_{{\mathrm{PC}}_{0}}}\times \frac{\left[\mathrm{PC}\right]}{\Delta t}\times \frac{1}{\text{cell}}$$

where the terms *A*_PC_, *A*_PC0_ and [*PC*] correspond to the values of carbon in this equation. The concentration of total inorganic carbon (DIC) was determined using a Shimadzu TOC‐VCSH. Inorganic carbon A _CO2_ was measured by injection of a sample into 25 wt% H_3_PO_4_ in the reaction vessel, evolving CO_2_.

### Calculations of C and N sources and demand

The C and N requirements of *B. bigelowii* were calculated using the C and N content per cell, growth rate and the C and N_2_ fixation rates per cell. The cellular C and N content were determined using the same samples that were analysed for their isotope compositions of ^13^C and ^15^N. This analysis was carried out using an EA‐IRMS as detailed in the preceding section. The total C and N content of each sample was measured and subsequently divided by the number of cells quantified via flow cytometry. C and N requirements per day were calculated as follows:

(4) $$C\left(\text{or}\;N\right)\;\text{requirement}\;\mathrm{per}\;\mathrm{day}=\begin{array}{l} C\left(\text{or}\;N\right)\;\text{content}/\text{cell}\\ \times \left(1-e^{\mu_{B.\text{bigelowii}}}\right) \end{array}$$

The percentage biomass contributed by C and N_2_ fixation was calculated by the C and N_2_ fixation rates/cell/day divided by the C and N requirement per day.

(5) $$\begin{array}{l} \%\text{biomass contributed}\;\mathrm{by}\;C\left(\text{or}\;N_{2}\right)\;\text{fixation}\\ \;=\frac{C\left(\text{or}\;N_{2}\right)\;\text{fixation rates}\;{\text{cell}}^{-1}\;d^{-1}}{C\left(\text{or}\;N\right)\;\text{requirements}\;d^{-1}} \end{array}$$

C uptake from grazing was estimated from the bacteria cell sizes, volume: biomass ratio, grazing rates and trophic efficiency. The dimensions of 12 bacteria were measured using epifluorescence microscopy (Axio Imager 2, Zeiss, Dublin, CA, USA) at 630× magnification and the width and length of each cell were determined using Zeiss ZEN Digital Imaging for Light Microscopy software. This involved capturing images of the cells and using the software to measure the width and length of each cell at the cross‐section. These measurements were then used to calculate the biovolume using the formula:

(6) $$\text{Volume}=\pi \times W^{2}\times \left(\frac{L}{4}-\frac{W}{6}\right)$$

where *W* is the width at the cross‐section and *L* is the length (Hillebrand et al., 1999).

The biovolume was then converted to C biomass using the equation:

(7) $$m=197\times V^{0.46}$$

where *m* is the C biomass and *V* is the volume (Cermak et al., 2017).

For the estimation of minimum and maximum C and N uptake from grazing:

(8) $$\begin{array}{l} C\left(\text{or}\;N\right)\;\text{uptake from grazing}\\ \;=m\times \text{Grazing Rate}\;d^{-1}\times \text{Trophic efficiency} \end{array}$$

with a conservative trophic transfer efficiency of 10%–30% (Priyadarshi et al., 2019).

Percentage biomass by grazing was calculated by:

(9) $$\%\text{biomass}=\frac{C\;\left(\text{or}\;N\right)\;\text{uptake from grazing}\;d^{-1}}{C\;\left(\text{or}\;N\right)\;\text{requirement}\;d^{-1}}$$

## RESULTS

**FIGURE 1:**
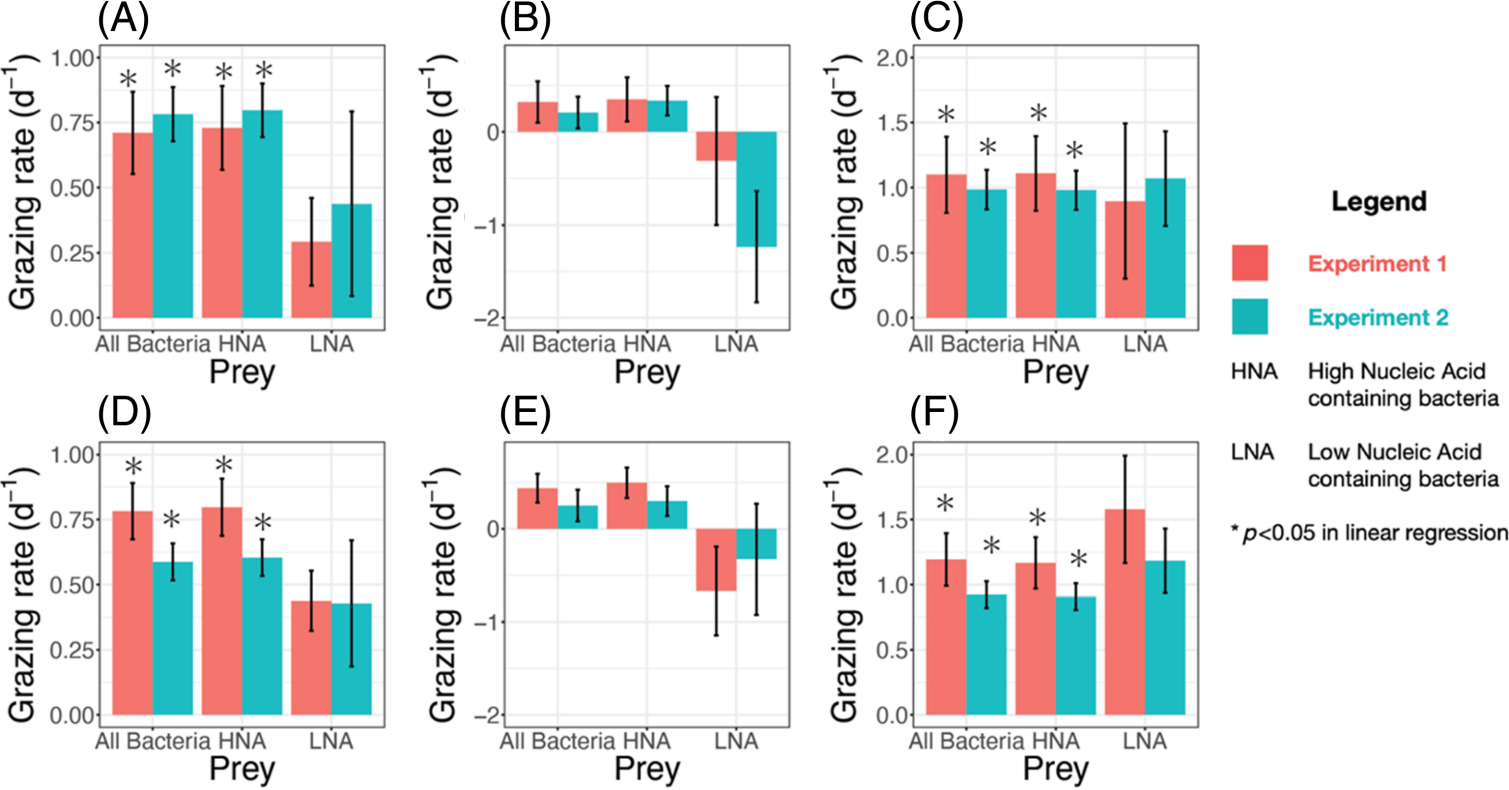
Grazing rates of *Braarudosphaera bigelowii* on co‐occurred bacterial prey and the intrinsic growth rates of the bacterial prey. A and D illustrate the total 24‐h grazing and growth rates, respectively. B and E detail the daytime grazing and growth rates, while C and F show the nighttime grazing and growth rates. The red and blue bars in each graph represent results from two replicate experiments. Statistically significant results (*p* < 0.05) for all 24‐h and nighttime rates are indicated by asterisks above the bars, although no significant grazing rates were observed for LNA‐containing bacteria, which should be interpreted with caution. HNA, high nucleic acid‐containing bacteria; LNA, LNA‐containing bacteria.

The results of the dilution experiments show that *B. bigelowii* was actively grazing on the co‐occurring bacteria in the culture. The three‐point dilution experiments were repeated twice and all treatments were done in triplicate. The 24‐h grazing rate of *B. bigelowii* on the co‐occurring bacterial population was determined to be 0.6–0.7 per day, which is the negative of the slope in the linear regression of bacterial growth rates against dilution levels (Figures 1A, S2A–C and S3A–C). This indicates the bacteria were being grazed at a rate of 6–7 cells/haptophyte/hour (Table S4). The intrinsic growth rate of the bacteria (μ_0_), when grazers are theoretically absent, was estimated as the *y*‐intercept in the linear regression and was 0.6–0.8/day (Figures 1D, S2A–C and 3A–C).

To compare grazing in the 12 h daytime period to the 12 h nighttime period, subsamples for cell counts were taken at the end of the light and dark cycles. The 12 h nighttime grazing rate ranged from 1.0 to 1.1/day (*p* = 0.009) (Figures 1C, S2D–F and 3D–F), which is equivalent to approximately 8 cells per hour, but there were no statistically significant grazing rates detected in the 12 h daytime period (Figures 1B, S2G–I and S3G–I). The 12 h nighttime intrinsic growth rate was 1.0–1.2/day (*p* = 0.009) (Figures 1F, S2D–F and S3D–F), however, the daytime intrinsic growth rate was not statistically significant from the linear regression (Figures 1E, S2G–I and S3G–I).

**FIGURE 2:**
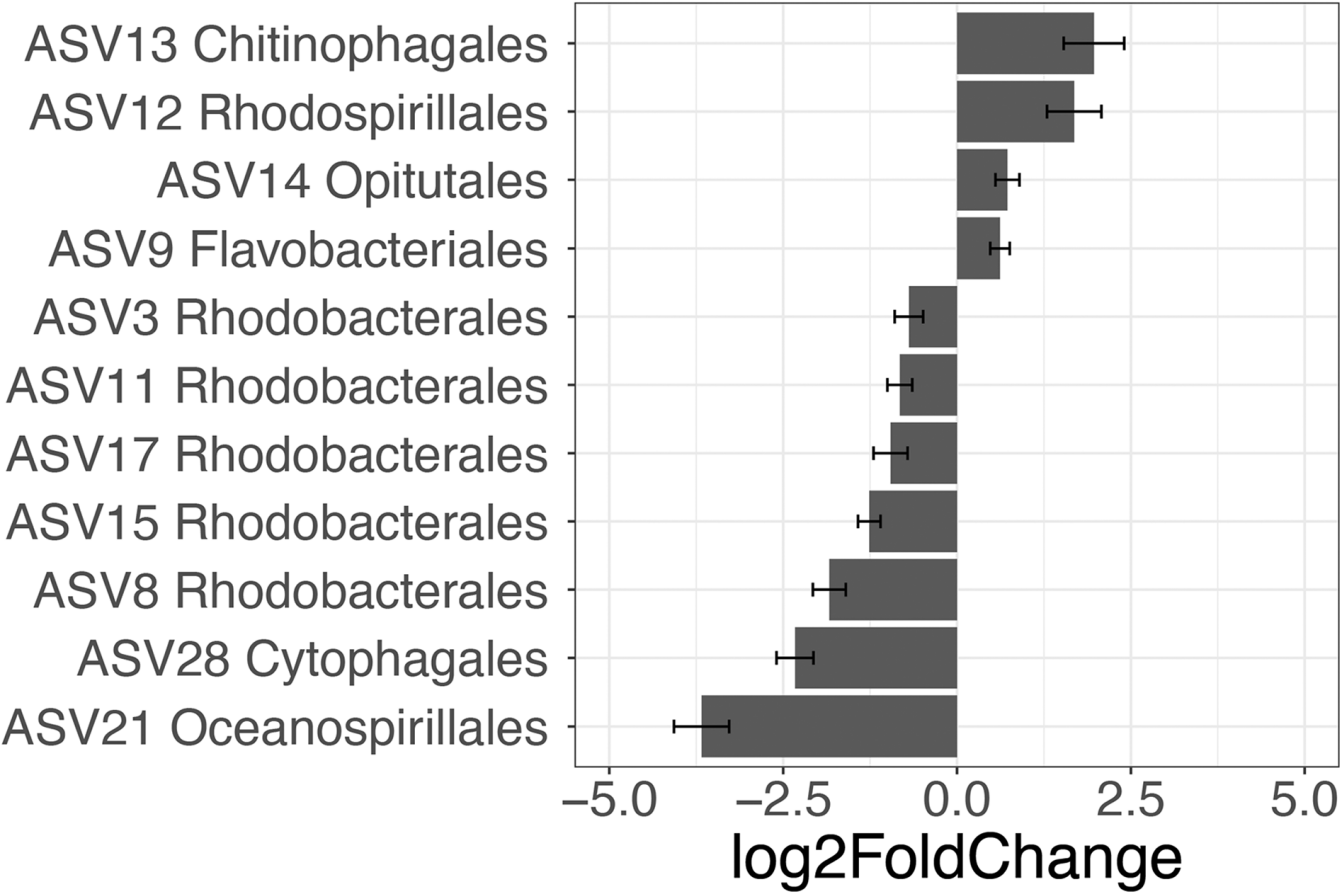
Changes in the relative abundances of amplicon sequence variants (ASVs) that showed differential changes in the 20% treatment within 24 h.

The composition of the bacteria assemblage was determined to detect whether *B. bigelowii* exhibits differential grazing on the co‐occurring bacterial species. The most abundant ASVs at T_0_ were in the orders Flavobacteriales (39.5%), followed by Alteromonadales (16.9%) and Rhodobacterales (15.1%) (Table S5). The relative abundances of some strains increased from T_0_ to T_24_ under the reduced grazing pressure in the 20% bottles, suggesting that these strains may be preferentially grazed. These strains included the orders Chitinophagales, Rhodospirillales, Flavobacteriales and Opitutales. The log2‐fold change was 0.61–1.96 (Figure 2). No statistically significant differences were observed between the 60% and 100% bottles throughout the incubation, despite the high grazing pressure in the 100% bottles. These changes may be attributed to differential grazing, differential bacterial growth, or a combination of both factors.

**FIGURE 3:**
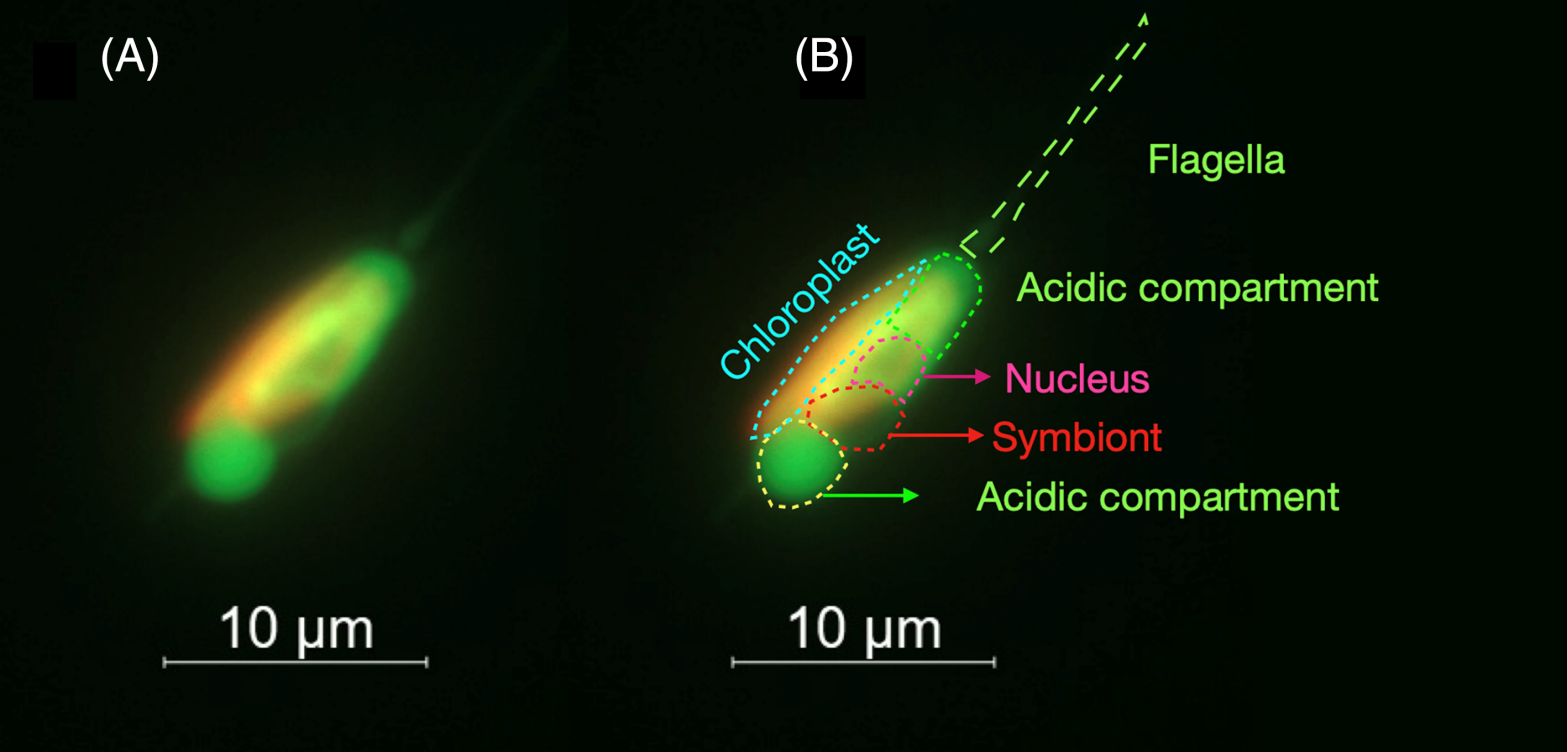
Fluorescent microscopy image of *Braarudosphaera bigelowii* after treatment with Lysotracker^TM^ Green DND‐26. The green fluorescence signal indicates acidic subcellular regions stained with Lysotracker^TM^ Green DND‐26 and chloroplasts are exited using red fluorescence. A and B show images of the same cell, with B labelled to highlight different components of the cell.

**FIGURE 4:**
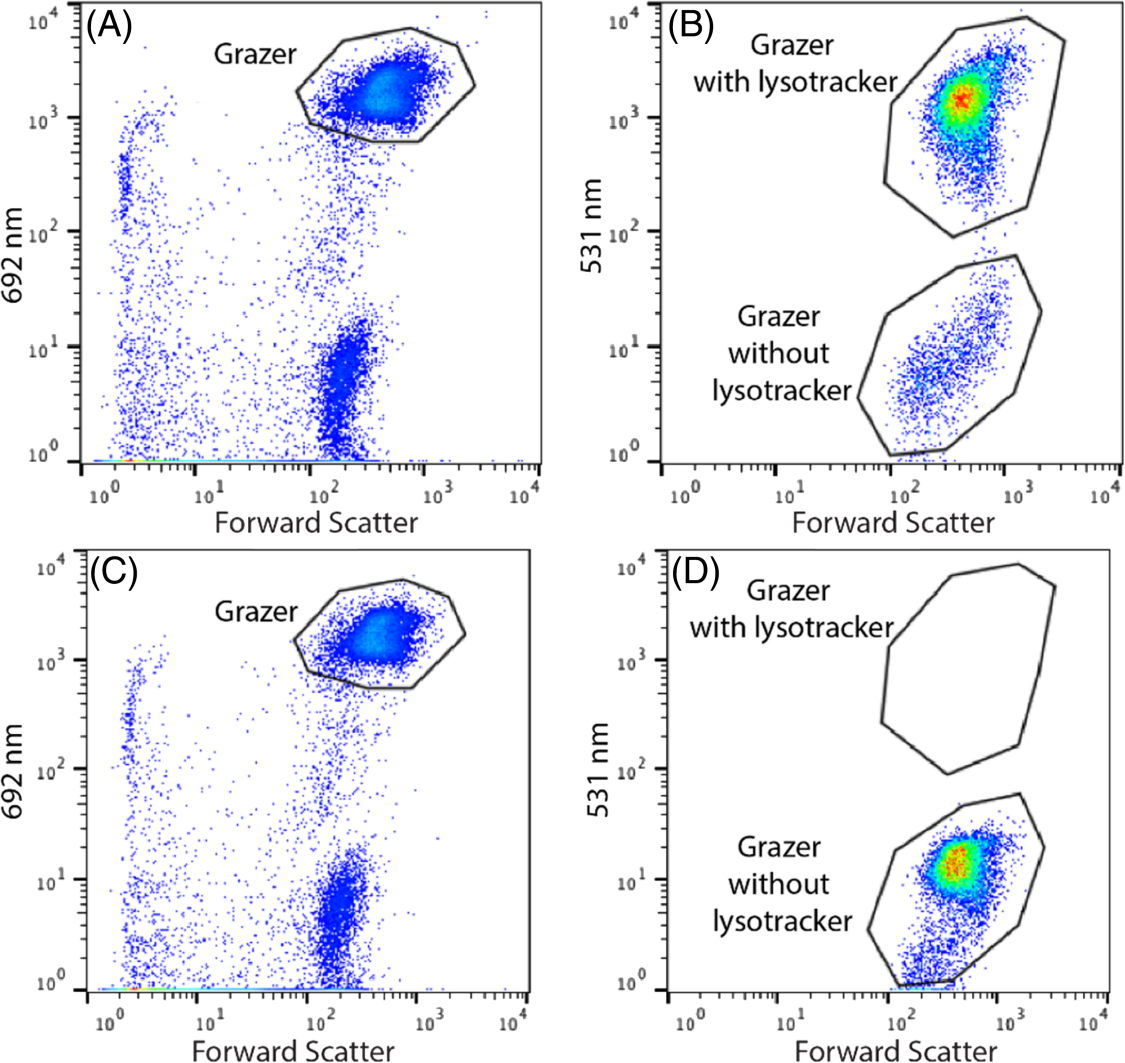
Cytograph depicting *Braarudosphaera bigelowii* stained with Lysotracker DND‐26. Panels (A, B) display the staining of Lysotracker DND‐26 at *B. bigelowii* chlorophyll signal in the 692 nm channel and Lysotracker DND‐26 staining in the 531 nm channel, respectively. Panels (C, D) serve as negative controls, showing the presence of *B. bigelowii* chlorophyll signals in the 692 nm channel and the absence of Lysotracker staining in the 531 nm channel, respectively.

*Braarudosphaera bigelowii* was observed under a microscope after staining with Lysotracker^TM^ Green DND‐26 to determine the digestive activity of the food vacuole. The vacuole surrounding UCYN‐A2 and the nucleus fluoresced green under 504/511 nm excitation/emission after staining. The brightest signal originated from the compartment closest to UCYN‐A2, but it also stained the compartment at the flagellar side of the cell (Figure 3A,B). This observation suggests the possibility of multiple vacuoles, yet it remains unclear whether these represent two distinct vacuoles or different regions of a single vacuole. The results were supported with flow cytometry enumeration of lysotracker‐stained cells, which showed that approximately 90% of the cells had green fluorescence (Figure 4A,B). No signal was observed in the same green fluorescing region in the unstained *B. bigelowii* culture (Figure 4C,D). The results indicate that there was recent digestive activity in the food vacuole that caused acidic conditions.

**FIGURE 5:**
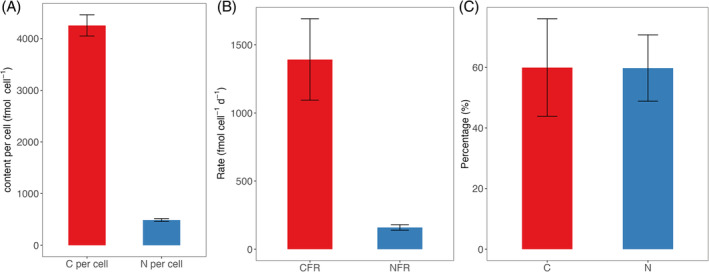
Summary of cellular growth, fixation rates and elemental content for *Braarudosphaera bigelowii*. Panel (A) quantifies the carbon and nitrogen content per cell. Panel (B) illustrates the carbon and nitrogen fixation rates per cell. Panel (C) depicts the percentage of cellular biomass attributable to carbon and nitrogen fixation.

C and N_2_ fixation and growth rates of *B. bigelowii* were measured to determine whether autotrophy was sufficient for the growth requirements. The growth rate of *B. bigelowii* under cultured conditions averaged 0.44 ± 0.03/day. The C fixation rate was 1393 ± 298 fmol C/cell/day and the C content was 4256 ± 206 fmol C/cell/day (Figure 5A,B). The percentage of total per cell C content obtained from C fixation was estimated to be 60 ± 16% (Figure 5C). The N_2_ fixation rate was 160 ± 20 fmol N/cell/day and the N content was 489 ± 27 fmol N/cell (Figure 5A,B). The percentage of total per cell N content obtained from N_2_ fixation was estimated to be 60 ± 11% (Figure 5C). The average co‐occurred bacteria biomass per cell was estimated to be ca. 76 fg C/cell, with the interquartile range as 23 fg C/cell. Measured grazing rates were 6.1–7.1/h (or 146 cells/day, assuming constant grazing rates throughout a diel cycle) and using a very conservative or trophic transfer efficiency from 10% to 30%, grazing contributes between 4.0% and 19% of the C biomass requirement and 6.7%–20% of N biomass requirement (Table 1).

**TABLE 1:** Potential grazing contribution of grazing to the daily growth rate requirements of *Braarudosphaera bigelowii*.

Estimates	Lower estimate	Higher estimate
Length of the bacteria (*L*) (μm)	2.36	2.73
Width of the bacteria (*W*) (μm)	0.228	0.296
Volume (μm^3^)	0.0900	0.174
Biomass (fg C/cell)	9530	15,100
Trophic efficiency (%)	10	30
% C biomass from grazing (%)	4.0	19
% N biomass from grazing (%)	6.7	20

*Note*: The lower and upper estimates of the cell length and width of the bacteria were determined using the lower quartile and upper quartile of the measured cells. Volumes were calculated following the methods outlined by Hillebrand et al. (1999). Biovolume was converted to biomass using the equation from Cermak et al. (2017) and the percentage of biomass from grazing was calculated based on trophic efficiency (Priyadarshi et al., 2019).

## DISCUSSION

### *Phagotrophy in* B. bigelowii *may provide an ecological advantage*

Our results provide multiple lines of evidence that support the hypothesis that *B. bigelowii* is capable of phagotrophy. Results from the dilution experiments demonstrate the haptophyte's ability to graze on prey cells, which is further supported by the lysotracker staining revealing the presence of an active food vacuole for digestion. It should be noted that while lysotracker is commonly used to specifically label acidic food vacuoles, it may also stain other acidic structures. Nonetheless, the presence of lysotracker‐labelled compartments and the measured grazing rates supports the phagotrophic activity of *B. bigelowii*. The C and N budget analysis reveals that the rates of photosynthesis and N_2_ fixation alone are insufficient to sustain the daily growth rates, indicating the reliance on an external nutritional source. The ability to utilize a phagotrophic mode of nutrition may confer an ecological advantage in different environmental conditions.

The nitrogen‐fixing *B. bigelowii/UCYN‐A* is one of the most abundant and widespread marine diazotrophs. Unlike other N_2_‐fixers that are typically associated with oligotrophic open ocean conditions, it can be found in the open ocean, temperate coastal regions, brackish water and even cold high‐latitude waters (Bentzon‐Tilia et al., 2015; Cheung et al., 2020; Harding et al., 2018; Shiozaki et al., 2020) and even actively transcribing *nifH* in coastal sediments (Brown & Jenkins, 2014). Based on our findings, it is plausible to interpret that the utilization of different nutritional modes may confer a distinct ecological advantage, enabling it to inhabit regions of the ocean with varying environmental properties. The ability to obtain fixed N from UCYN‐A is a potential ecological advantage in oligotrophic ocean regions and the photosynthetic capability of *B. bigelowi* *i* allows UCYN‐A to harvest light energy from the euphotic zone. In regions where light is limited, such as in high latitude waters (which are seasonally‐light limited), coastal waters and sediments and the lower euphotic zone, the phagotrophic capability of *B. bigelowii* may provide a means to acquire supplementary energy from grazing. The phagotropic lifestyle allows *B. bigelowii* to adapt to various ocean regimes, potentially explaining its presence in the oligotrophic open ocean, coastal and high‐latitude regions.

*Braarudosphaera bigelowii* exhibited a pattern that suggests possible selective grazing on certain bacterial strains, as evidenced by the increase of abundances of certain strains when grazing pressure was decreased. Specifically, Chitinophagales, Rhodospirillales, Flavobacteriales and Opitutales exhibit enhanced abundance under reduced grazing pressure. Of the four orders that were preferentially grazed, three orders – Chitinophagales, Flavobacteriales and Opitutales were classified as copiotrophs capable of degrading diverse organic compounds, such as chitin and cellulose (Rosenberg et al., 2014), fermenting carbohydrates (Chin et al., 2001) and breaking down phosphoric acid monoesters to phosphate (Rosenberg et al., 2014). While these findings are intriguing, they should be interpreted with caution. It should be noted that our culturing conditions might have enriched copiotrophs due to the presence of organic carbon (GJE). As a result, the observed copiotrophs here may not necessarily represent the bacterial population that exists in the marine environments where *B. bigelowii* is found. Typically, copiotrophs thrive in nutrient‐rich ‘hot spots’ and may not be as prevalent in the oligotrophic open ocean, suggesting that our findings are potentially more applicable to nutrient‐replete areas. An additional consideration is the ‘bottle effect,’ referring to changes in microbial population dynamics that can occur within confined culture systems over time. Such effects could contribute to the observed shifts in bacterial composition independently of grazing pressures. Therefore, while differential grazing is a plausible driver of the changes in bacterial abundance we observed, we cannot exclude other contributing mechanisms and the definitive impacts of our findings remain to be fully elucidated.

### Carbon and nitrogen requirements for growth may be supported by grazing

Our findings indicate that phagotrophy is important to supplement energy in *B. bigelowii* under these culture conditions. In nutrient‐replete conditions where the culture is maintained, the estimated C and N requirements for the growth of *B. bigelowii* could not be met solely by C and N_2_ fixation. Only 60 ± 16% of the total C quota and 60 ± 11% of the total N quota needed to sustain the measured growth rates were supplied by CO_2_ and N_2_ fixation. This suggests that *B. bigelowii* needed external C and N sources to maintain growth. While it is difficult to accurately estimate the trophic efficiency, using the conservative estimates of both trophic efficiency and bacterial biomass, grazing could account for 4.0% to 19% of the carbon requirements of *B. bigelowii*. However, it is important to note that the bacterial concentrations in our cultures are one to two orders of magnitude higher than those typically found in coastal marine environments, where concentrations typically range from approximately 10^5^ to 10^7^ cells/mL (Whitman et al., 1998). This discrepancy is worth noting because grazing rates generally increase with prey concentration (Holling, 1959). Therefore, the grazing rates observed in this study may be higher than those occurring under natural conditions.

The combined contributions of photosynthesis, N_2_ fixation and phagotrophy are insufficient to meet the daily C and N requirements of *B. bigelowii*, indicating its potential reliance on additional nutritional sources beyond autotrophy and phagotrophy. It is speculated that *B. bigelowii* may engage in osmotrophy, potentially uptaking DOM provided by the addition of GJE or produced by co‐occurring bacteria to meet the C and N requirements. However, the exact role and necessity of DOM in supporting growth remain speculative and recent reports suggest that *B. bigelowii* can also grow without GJE under different laboratory conditions (Tyler Coale, unpublished). This suggests that *B. bigelowii* may have adaptable strategies for nutrient acquisition in various marine environments. For instance, in the theoretical absence of DOM, *B. bigelowii* might increase its autotrophic or grazing activities to compensate for the decrease in essential nutrients. Conversely, a decrease in autotrophic or grazing activities might also occur due to the unavailability of critical compounds typically supplied by DOM, impacting the growth of *B. bigelowii*. Further research is required to disentangle these factors and to determine whether phagotrophy in *B. bigelowii* is obligatory or facultative and to clarify the role of grazing in environments where bacteria are scarce or DOM is limited.

Similarly, N_2_ fixation could supply only 60 ± 11% of *B. bigelowii* N requirement, which suggests there is another N source. By employing a similar estimate for trophic transfer and assuming a C:N ratio of 5.9 (Fukuda et al., 1998), grazing could account for 6.7%–20% of the N requirement. However, this estimate should be approached with caution, as the trophic efficiency of N is not well‐documented or measured and the C:N ratio of the bacteria was estimated based on ocean values, not directly measured. A recent transcriptome study of *B. bigelowii* revealed the absence of many transcripts relating to the utilization of common N substrates, such as nitrate, nitrite, formamide, glutamate, glutamine, cyanate and urea (Suzuki et al., 2021). Although this could be due to the nature of the transcriptomic study which may be missing some information due to the lack of the complete genome, another reason could be that *B. bigelowii* were not utilizing these N sources despite their presence in the growth medium. An environmental study of *B. bigelowii* by Mills et al. (2020) using nanoSIMS showed that it could not take up nitrate as an N source, but could take up some ammonium. This result was also supported by the transcriptomic study that showed that the ammonium transporters were expressed (Suzuki et al., 2021). However, the ammonium transporter gene expressed could be due to the need of *B. bigelowii* to acquire ammonium from the UCYN‐A body, which produces ammonium as a product of N_2_ fixation. Suzuki et al. (2021) suggested some co‐occurring bacteria were capable of reducing nitrate to ammonium and the ammonium transporter gene in *B. bigelowii* could be expressed to the uptake of ammonium by diffusion, or by grazing (Suzuki et al., 2021). Our experimental results also agreed with the metatranscriptomic‐based analysis by Suzuki et al. (2021) that *B. bigelowii* was predicted to be highly likely a phagotroph.

*Braarudosphaera bigelowii* was shown to have the highest grazing rates during the night and cease grazing during the day. The reason for nocturnal grazing remains highly speculative. However, it is important to consider that initiating experiments during daylight hours could offer a clearer understanding of whether the observed cessation of grazing activities in daylight is due to a saturation of grazing or an influence of circadian rhythms on grazing behaviour. Such alternative experimental setups could help disentangle the effects of experimental initiation from the natural grazing dynamics of *B. bigelowii*. Nonetheless, an interpretation drawn from these findings suggests that *B. bigelowii*, by incorporating carbon and nitrogen through photosynthesis and N_2_ fixation during the daytime, could engage in night grazing to replenish carbon and nitrogen levels along with other essential nutrients. This nocturnal grazing pattern might align more effectively with the cell's energy dynamics, allocating ATP and reductants primarily for nitrogen fixation during the day. On the other hand, cell division (Landa et al., 2021), grazing and digestion activities predominantly occur during the nighttime. Jones (1997) classified mixotrophy into four categories, according to the degree of phagotrophy and phototrophy in different kinds of mixotrophs. Group A predominantly uses phagotrophy, while groups B, C and D rely primarily on phototrophy as their main mode of nutrition. In group B, the ingestion rates are inversely proportional to light intensity, whereas group C uses phagotrophy to acquire essential nutrients and thus grazing rates are proportional to light intensity, resulting in feeding during the daytime. Group D represents an extreme case where ingestion of prey only occurs during prolonged periods of darkness (Jones, 1997). *Braarudosphaera bigelowii* exhibits characteristics that are most similar to group B mixotrophs. *Braarudosphaera bigelowii* mainly obtains its C through photosynthesis. A majority of C needed for growth (60 ± 16%) is obtained through phototrophy indicating it is the primary mode of nutrition. Grazing occurs at night when light is absent, suggesting that the main goal of feeding is to supplement energy as an alternative carbon source. However, the *B. bigelowii* culture could not be grown axenically, suggesting that interactions or nutrients associated with other microorganisms are necessary for growth. Phagotrophy might become advantageous when nutrients are scarce. An example of this group is the haptophyte *Chrysochromulina brevifilum*, where ingestion rates are inversely proportional to light intensity and grazing is utilized to supplement energy in conditions of limited light availability, thus allowing for optimal growth (Hansen & Hjorth, 2002). *Braarudosphaera bigelowii* exhibits a similar mixotrophic lifestyle but with diazotrophic capabilities, allowing it to thrive in a variety of oceanic environments and making it a unique and important organism in marine ecosystems.

## CONCLUSIONS

Our study provides evidence that the *B. bigelowii* is a phagotroph, by measuring grazing rates of *B. bigelowii* on co‐occurring bacteria in dilution experiments. The results indicate *B. bigelowii* grazes at night, likely for supplemental nutrition. *Braarudosphaera bigelowii* demonstrated preferential grazing towards certain groups of copiotrophs and maintained an active food vacuole for digestion. Taken together, our study provides novel insights on how *B. bigelowii* switches between different modes of nutrition to maintain its C and N requirement in varying environmental conditions, which may explain its broad environmental distribution and persistence in light‐limited habitats.

## AUTHOR CONTRIBUTIONS

**Esther Wing Kwan Mak:** Data curation (lead); formal analysis (lead); investigation (lead); methodology (lead); visualization (lead); writing – original draft (lead); writing – review and editing (lead). **Kendra A. Turk‐Kubo:** Conceptualization (equal); investigation (supporting); methodology (supporting); writing – review and editing (equal). **David A. Caron:** Formal analysis (equal); methodology (equal); writing – review and editing (equal). **Rachel C. Harbeitner:** Data curation (equal); investigation (equal); methodology (equal); writing – review and editing (equal). **Jonathan D. Magasin:** Formal analysis (equal); investigation (equal); methodology (equal); software (equal); writing – review and editing (equal). **Tyler H. Coale:** Data curation (equal); investigation (equal); methodology (equal); writing – review and editing (equal). **Kyoko Hagino:** Methodology (equal); resources (equal); writing – review and editing (equal). **Yoshihito Takano:** Methodology (equal); resources (equal); writing – review and editing (equal). **Tomohiro Nishimura:** Methodology (equal); resources (equal); writing – review and editing (equal). **Masao Adachi:** Methodology (equal); resources (equal); writing – review and editing (equal). **Jonathan P. Zehr:** Conceptualization (equal); funding acquisition (lead); supervision (lead); writing – review and editing (lead).

## CONFLICT OF INTEREST STATEMENT

The authors declare that there are no conflicts of interest.

## Supporting information


**Data S1.** Supporting information.

## Data Availability

The data that supports the findings of this study are available in the supplementary material of this article.
