# Supplementary material for: Phagotrophy in the nitrogen‐fixing haptophyte Braarudosphaera bigelowii
Source: Environ Microbiol Rep. 2024 Jul 24;16(4):e13312. doi: 10.1111/1758-2229.13312 (PMC11269211; doi:10.1111/1758-2229.13312)
Supplement: Supplementary file 1 — Data S1. Supporting information. [file EMI4-16-e13312-s001.docx]

**Supplemental figure 1.** Temporal growth patterns of the haptophyte. This figure illustrates the growth curve of the haptophyte over time, with cell concentration plotted against the day. The establishment of a stable growth rate is evident prior to the measurement of specific growth rates. The reductions in cell concentration observed on days -3 and 0 correspond to the culture transfers to more dilute conditions, which were necessary to maintain semi-continuous growth. Each line represents one of three replicate cultures, color-coded as follows: red (Replicate A), blue (Replicate B), and green (Replicate C), allowing for the comparison of growth consistency across replicates.

**Supplemental figure 2.** Growth rates of different bacterial groups across dilution levels and different durations of the day for experiment 1. Panels (a, d, g) show the growth rate of total bacteria, panels (b, e, h) depict the growth rate of high nucleic acid (HNA) bacteria, and panels (c, f, i) represent the growth rate of low nucleic acid (LNA) bacteria. Each panel corresponds to different experimental conditions: 24-hour total (a, b, c), nighttime (d, e, f), and daytime (g, h, i) periods. The linear regression lines (in blue) indicate the trend of growth rate changes with respect to the dilution levels. The equations of the linear regression and the coefficient of determination (R^2^) values are also presented in blue, providing a quantitative measure of the strength of the relationship between the bacterial growth rates and dilution levels for each time frame.

**Supplemental figure 3.** Growth rates of different bacterial groups across dilution levels and different durations of the day for experiment 2. Panels (a, d, g) show the growth rate of total bacteria, panels (b, e, h) depict the growth rate of high nucleic acid (HNA) bacteria, and panels (c, f, i) represent the growth rate of low nucleic acid (LNA) bacteria. Each panel corresponds to different experimental conditions: 24-hour total (a, b, c), nighttime (d, e, f), and daytime (g, h, i) periods. The linear regression lines (in blue) indicate the trend of growth rate changes with respect to the dilution levels. The equations of the linear regression and the coefficient of determination (R^2^) values are also presented in blue, providing a quantitative measure of the strength of the relationship between the bacterial growth rates and dilution levels for each time frame.

**Supplementary table 4. Table showing the grazing rates per grazer calculated from two dilution experiments.**

**Supplementary table 4a. Table showing the grazing rates (Experiment 1)**

Total Bacteria HNA LNA

Bacteria µ with grazer 0.11 0.12 0.01

Bacteria µ with grazer 0.12 0.12 0.04

Bacteria µ with grazer 0.12 0.14 -0.05

Bacteria µ without grazer 1.25 1.27 0.94

Duration 24h 24h 24h

Grazer concentration mean 1.6E05 1.6E05 1.6E05

Bacteria concentration mean 2.4E07 2.2E07 2.0E06

Ingestion Rate (cells/grazer/h) 7.16 6.61 0.49

Ingestion Rate (cells/grazer/h) 7.12 6.59 0.47

Ingestion Rate (cells/grazer/h) 7.09 6.51 0.52

Mean 7.12 6.57 0.49

Stdev 0.03 0.05 0.02

**Supplementary table 4b. Table showing the grazing rates (Experiment 2)**

Total Bacteria HNA LNA

Bacteria µ with grazer 0.16 0.16 0.04

Bacteria µ with grazer 0.16 0.7 0.11

Bacteria µ with grazer 0.17 0.16 0.41

Bacteria µ without grazer 0.78 0.80 0.44

Duration 24h 24h 24h

Grazer concentration mean 3.8E05 3.8E05 3.8E05

Bacteria concentration mean 9.0E07 8.6E07 4.4E06

Ingestion Rate (cells/grazer/h) 6.18 5.97 0.19

Ingestion Rate (cells/grazer/h) 6.09 5.91 0.16

Ingestion Rate (cells/grazer/h) 6.03 6.02 0.01

Mean 6.10 5.97 0.12

Stdev 0.07 0.05 0.09

**Supplemental table 5. Taxonomic distribution of the top 14 amplicon sequence variants (ASVs).** This table summarizes the relative abundances and taxonomic classifications of the top 14 ASVs identified from 16S rRNA gene sequencing. The ASVs are presented along with their corresponding percentage of the total sequence count and taxonomic hierarchy, ranging from kingdom to species level, where available. ASVs are ranked by their relative abundance within the sampled microbial community.

ASV Percentage Kingdom Phylum Class Order Family Genus Species

ASV1 39.53 Bacteria Bacteroidetes Bacteroidia Flavobacteriales Flavobacteriaceae Tenacibaculum NA

ASV2 16.87 Bacteria Proteobacteria Gammaproteobacteria Alteromonadales Alteromonadaceae Alteromonas NA

ASV3 8.00 Bacteria Proteobacteria Alphaproteobacteria Rhodobacterales Rhodobacteraceae Tropicibacter phthalicicus

ASV4 7.07 Bacteria Proteobacteria Alphaproteobacteria Rhodobacterales Rhodobacteraceae NA NA

ASV5 4.93 Bacteria Cyanobacteria Oxyphotobacteria Chloroplast NA NA NA

ASV6 3.80 Bacteria Cyanobacteria Oxyphotobacteria Nostocales Microcystaceae Atelocyanobacterium_(UCYN-A) thalassa

ASV7 3.34 Bacteria Proteobacteria Alphaproteobacteria Rhodobacterales Rhodobacteraceae NA NA

ASV8 2.35 Bacteria Proteobacteria Alphaproteobacteria Rhodobacterales Rhodobacteraceae Thalassobius NA

ASV9 2.00 Bacteria Bacteroidetes Bacteroidia Flavobacteriales Cryomorphaceae NA NA

ASV10 1.84 Bacteria Proteobacteria Alphaproteobacteria Caulobacterales Hyphomonadaceae Maricaulis maris

ASV11 1.77 Bacteria Proteobacteria Alphaproteobacteria Rhodobacterales Rhodobacteraceae Sulfitobacter geojensis

ASV12 1.07 Bacteria Proteobacteria Alphaproteobacteria Rhodospirillales Terasakiellaceae NA NA

ASV13 0.84 Bacteria Bacteroidetes Bacteroidia Chitinophagales NA NA NA

ASV14 0.72 Bacteria Verrucomicrobia Verrucomicrobiae Opitutales NA NA NA
